# Acute Interstitial Pneumonia (Hamman-Rich Syndrome) as a Cause of Idiopathic Acute Respiratory Distress Syndrome

**DOI:** 10.1155/2011/628743

**Published:** 2011-05-29

**Authors:** Jackrapong Bruminhent, Shahla Yassir, James Pippim

**Affiliations:** Department of Internal Medicine, St. Vincent's Medical Center, 2800 Main Street, Bridgeport, CT 06606, USA

## Abstract

Hamman-Rich syndrome, also known as acute interstitial pneumonia, is a rare and fulminant form of idiopathic interstitial lung disease. It should be considered as a cause of idiopathic acute respiratory distress syndrome. Confirmatory diagnosis requires demonstration of diffuse alveolar damage on lung histopathology. The main treatment is supportive care. It is not clear if glucocorticoid therapy is effective in acute interstitial pneumonia. We report the case of a 77-year-old woman without pre-existing lung disease who initially presented with mild upper respiratory tract infection and then progressed to rapid onset of hypoxic respiratory failure similar to acute respiratory distress syndrome with unknown etiology. Despite glucocorticoid therapy, she did not achieve remission and expired after 35 days of hospitalization. The diagnosis of acute interstitial pneumonia was supported by the histopathologic findings on her lung biopsy.

## 1. Introduction

Acute interstitial pneumonia (AIP) or Hamman Rich syndrome is a rare and fulminant form of lung injury, originally described by Hamman and Rich in 1935 [1]. It is an interstitial lung disease characterized by rapid onset of respiratory failure, similar to acute respiratory distress syndrome (ARDS) with diffuse alveolar damage (DAD) on lung biopsy specimens. 

While the mechanism of the interstitial pneumonia remains elusive, recent studies have suggested possible pathogenetic mechanisms. Specifically, both natural killer cells and chemokines such as interleukin-18 and interleukin-2 may play important roles in the evolution of acute cell injury into unremitting fibrosis specifically through abnormal wound repair [2].

The clinical presentation of AIP, as noted in several case series, has been reported in the literature [3–6]. The onset of the disease is usually abrupt, with a prodromal illness that lasts 1 to 2 weeks prior to presentation [6, 7]. The most common clinical symptoms are fever, cough, and shortness of breath [6]. Further, AIP is characterized by the rapid development of acute respiratory failure in a previously healthy individual without a history of lung disease. It is not associated with cigarette smoking and occurs with roughly equal frequency in men and women. The majority of patients are between 50 and 55 years of age [3, 6, 7].

Plain chest radiographic studies of AIP reveal a diffuse, bilateral, air-space opacification pattern, and high-resolution-computed tomography (HRCT) of the chest shows bilateral, patchy, symmetric areas of ground glass attenuation. Thus, AIP closely resembles ARDS both clinically and radiologically [8]. In fact, the current accepted criteria for the diagnosis of AIP include (1) a clinical syndrome of idiopathic ARDS and (2) pathologic confirmation of organizing DAD. Therefore, an open or thoracoscopic lung biopsy is required to confirm the diagnosis. 

According to the American Thoracic Society and European Respiratory Society International Multidisciplinary Consensus Classification of the Idiopathic Interstitial Pneumonias, Lung biopsies from patients with AIP typically shows diffuse involvement, although there may be variation in the severity of the changes among different histologic fields. The exudative phase shows edema, hyaline membranes, and acute interstitial inflammation. The organizing phase shows loose organizing fibrosis, mostly within alveolar septa and type II pneumocyte hyperplasia [9]. 

Generally, the primary focus of therapy is supportive care including supplemental oxygen and ventilatory support. Several reports have reported benefit from the use of glucocorticoids, in the treatment of AIP, but others contradict this finding [6]. Alternative immunosuppressive therapies (e.g., vincristine, cyclophosphamide, cyclosporine, and azathioprine) and lung transplantation have been reported in case series of AIP, with limited success [3, 10, 11].

Even with intensive treatment, including mechanical ventilation, the mortality from AIP remains high (>60 percent), and the majority of patients die within six months of presentation [6]. Notably, survivors of AIP did not experience recurrence and enjoyed complete or near complete recovery of lung function [12, 13]. 

Here, we describe a fatal case of AIP, progressing to acute hypoxic respiratory failure. Notably, the disease progression was not reversed by either mechanical ventilation or intravenous steroid therapy, and the patient expired after 35 days of hospitalization.

## 2. Case Presentation

**Figure 1:**
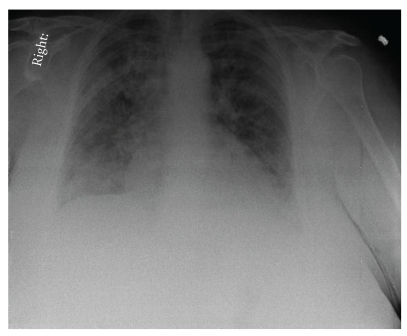
Chest radiograph showed acute bilateral pulmonary infiltration, more confluent in the areas of the right upper and bilateral lower lobes.

**Figure 2:**
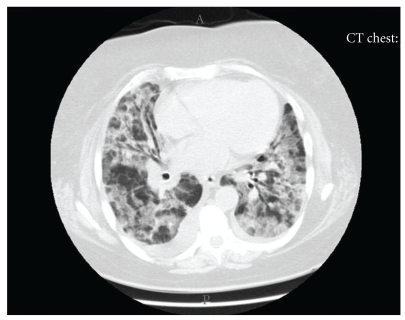
High resolution CT of the chest showed diffuse ground glass opacities mainly involving the upper lobes.

**Figure 3:**
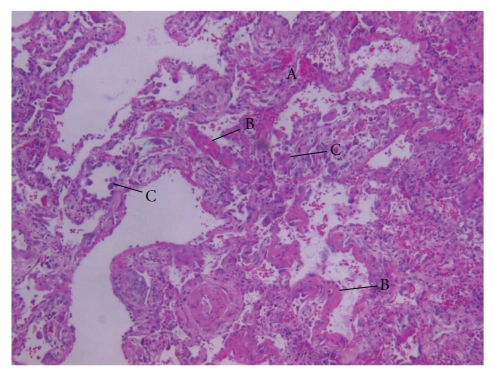
Histopathology showed diffuse alveolar damage pattern-mixed exudative and organizing phase, as demonstrated by video-assisted thoracostomy lung biopsy: showing interstitial edema and hemorrhage (A), diffuse alveolar wall thickening by proliferating connective tissue, formation of hyaline membranes, (B) and type II pneumocyte hyperplasia (C), (hematoxylin- eosin stain) (original × 100).

A 77-year-old woman with a past medical history of diabetes mellitus type 1, polymyalgia rheumatica, gastroesophageal reflux disease and hypertension, was brought to the emergency department after falling on the floor, without loss of consciousness. This was preceded by sore throat and lethargy for 3 days. Her symptoms were associated with a slight cough but she denied fever, chills, or shortness of breath. Upon presentation, she was afebrile, without tachypnea, or oxygen desaturation on room air. Lung examination revealed scattered rhonchi bilaterally. Chest radiograph showed no obvious pulmonary disease. She was admitted with an initial diagnosis of near syncope and mild upper respiratory infection. She was treated with azithromycin intravenously, with anticipated discharge home in 24 to 48 hours. After 48 hours of hospitalization, her clinical course was complicated by sudden onset shortness of breath and hypoxemia of unclear etiology. A repeat chest radiograph revealed acute wide spread pulmonary infiltrates, which represented a significant change from the prior study (Figure 1). Diuretics were started with a presumptive diagnosis of congestive heart failure. However, because she had a normal B-type natriuretic peptide level, and normal echocardiogram, a primary pulmonary process was suspected. High-resolution computed tomography (HRCT) of the chest revealed diffuse ground glass opacities throughout the lung fields, with bilateral traction bronchiectasis (Figure 2). Video-assisted thoracoscopic surgery with lung biopsy was performed on hospital day 19. The lungs revealed diffuse alveolar wall thickening with proliferating connective tissue, formation of hyaline membranes, and type II pneumocyte hyperplasia (Figure 3) compatible with DAD pattern: mixed exudative and organizing phase. Microbiologic investigations for infectious pathogens, including those on lung biopsy specimen, were negative. She was transferred to intensive care unit secondary to severe acute hypoxic respiratory failure, Pao2/Fio2 ratio of 89, because she could not be extubated after the procedure. She received ventilation in the volume assist-control mode, with a positive-end expiratory pressure (PEEP) of 5–8 cm H_2_O and tidal volume of 6 mL per measured body weight. Intravenous methylprednisolone 60 mg every 6 hours was given for the treatment of AIP, but the steroids dose was later tapered down. Despite initiation of intravenous glucocorticoid and high concentration of oxygen, she remained intubated for 5 weeks. Her clinical situation deteriorated further with the development of sepsis and acute kidney injury, and she eventually expired after 35 days of hospitalization.

## 3. Discussion

Our patient presented at an older age than most of the patients with AIP reported previously. She had the clinical and radiological profile of AIP. She had sudden onset and rapid progression of her symptoms, which helped to differentiate AIP from other forms of idiopathic interstitial pneumonia in which duration of symptoms is usually in months to years [3, 14]. HRCT of the chest revealed diffuse ground glass opacities and bronchial dilatation with architectural distortion, which are the most common findings [15]. The diagnosis was confirmed by the histological finding of DAD pattern seen on lung biopsy specimen. Infectious etiology was excluded on the basis of microbiological investigations. 

One recent study reported higher survival rates with early aggressive diagnostic approach, lung-protective mechanical ventilation, and the early institution of immunosuppressive therapy [16].

Our patient received lung-protective mechanical ventilation with low tidal volume and moderately high of PEEP. It is possible that the unsuccessful remission seen in our patient may have been influenced by the delayed diagnosis: 19 days compared to the mean duration from admission to diagnosis of 3.5 days from previous series which have been associated with higher survival rates [16]. Our patient received intravenous glucocorticoid, one of the recommended treatments [7], although there are no convincing data to support this practice [17]. Effectiveness of steroids is also probably dependent on early diagnosis, the extent of fibrosis, and the ratio of inflammation to fibrosis at the time of diagnosis. We believed that delayed treatment may not lead to a good clinical response and the addition of immunosuppressive therapy, such as cyclophosphamide or vincristine, is also not likely to affect the natural course of the late stage of the disease especially with extensive fibrosis [17]. 

Our patient was severely ill, with Pao2/Fio2 of 89: severe disease associated with a high mortality rate [3, 17]. Ichikado et al. noticed a close correlation between radiological findings and pathologic phases of DAD. They determined that patients who have the radiological findings of traction bronchiectasis, as seen in our patient, have more severe disease and a higher mortality [5, 18]. 

To date, there are no published guidelines on the management of AIP. In summary, despite mechanical ventilation, supportive care, and corticosteroid therapy, our patient did not achieve remission and expired within 35 days of hospitalization. This confirms the high mortality rate in AIP similar to that reported in previous series.

## 4. Conclusion

Sudden onset acute hypoxic respiratory failure in a patient without pre-existing lung disease should suggest the presence of interstitial lung disease. AIP should be considered in the differential diagnosis of ARDS, when the etiology remains unclear. The main treatment is supportive care. Mechanical ventilation is often required. Although early glucocorticoid or immunosuppressive therapy has been reported to improve the clinical outcomes, its efficacy is yet to be proven.

##  Competing Interests

The authors declare that they have no competing interests.

## Abbreviations

AIP: Acute interstitial pneumonia
ARDS: Acute respiratory distress syndrome
DAD: Diffuse alveolar damage
HRCT: High-resolution-computed tomography
PEEP: Positive-end expiratory pressure.
